# Supplementary material for: Role of TLRs in HIV-1 Infection and Potential of TLR Agonists in HIV-1 Vaccine Development and Treatment Strategies
Source: Pathogens. 2023 Jan 5;12(1):92. doi: 10.3390/pathogens12010092 (PMC9866513; doi:10.3390/pathogens12010092)
Supplement: Supplementary file 1 [file pathogens-12-00092-s001.zip › pathogens-2102094-supplementary.pdf]

Table S1. The selected list of TLR genetic polymorphisms and their association with HIV-infection and co-infections

GENE	SNP/ polymorphism	Study	Population	Susceptibility	Clinical findings
TLR1	rs5743551, rs5743618	Willie et al 2014 [55]	North America, Caucasian, Africans ancestry	SA with HIV-1 infection in Caucasians	NA
TLR1	GTGT	Willie et al 2014 [55]	North America, Caucasian, Africans ancestry	SA with HIV infection in Caucasians	NA
TLR1	ATGG	Willie et al 2014 [55]	North America, Caucasian, Africans ancestry	Protective against HIV in Caucasians	NA
TLR1	rs5743618, rs4833095	Varshney et al 2022 [82]	various	Association between TB disease in a particular ethnic population	The protection against TB
TLR2	rs111200466 196 -174 Ins/Del	Vidyant et al 2017 [56]	North Indian	SA with HIV-1 infection	Associated with reduced risk of disease progression
TLR2	rs111200466 196 -174 Ins/Del	Royo et al 2020 [57]	Spanish	Protective against HIV infection	NA
TLR2	rs111200466 196 -174 Ins/Del	Laplana et al 2020 [58]	Spanish	Associated with elevated risk of HIV infection	Associated with faster disease progression
TLR2	rs3804099 196 -174 Ins/Del	Shi et al 2020 [54]	various		Associated with HIV-1 infection clinical results
TLR2	2180 C/T, C/C, C/T	Kaushik et al 2022 [59]	North Indian	NS to HIV and TB	NS among different patient groups

TLR2	rs3804100<br>1350 C/C	Beima-Sofie et al 2013 [74]	Kenyan, infants	Associated with HIV-1 infection	Associated with time of mortality in female infants infected
TLR2	rs3804099<br>597T/C	Bochud et al 2007 [78]	Swiss	NA with protection against rapid progression	NA
TLR2_TLR6	TGTTG_GTCTCA	Willie et al 2014 [55]	North America, Caucasian, Africans	Protective against HIV, SA in Caucasians	NA
TLR2	rs3804099<br>-196 to 174 del	Varshney et al 2022 [82]	Various	association between TB disease in a particular ethnic population	The protection against TB
TLR3	rs3775291<br>1660 C/T	Huik et al 2013 [61]	Caucasian	Protective effect against HIV infection	NA
TLR3	rs3775296	Habibabadi et al 2020 [63]	Iranian	SA with HTLV-1 infection, mab be protective against HTLV-1 infection	NA
TLR4	rs4986790<br>896A/G	Vidyant et al 2019 [62]	Indian	Associated with HIV-1 infection	Linked with stage progression, not with CD4 count decrease, G allele higher in stage I
TLR4	rs4986790<br>896A/G	Jabłońska et al 2020 [66]	Caucasian, children and adolescents	Associated with the risk of infectious mononucleosis	Elevated liver enzyme levels and leukocytes
TLR4	rs4986790<br>896A/G	Kaushik et al 2022 [59]	North Indian	SI to active TB and HIV+ patients ($P < 0.001$)	SA among different groups
TLR4	rs4986790<br>1063A/G	Shi et al 2020 [54]	various	NA	Associated with HIV-1 infection clinical results
TLR4	rs4986791<br>1196C/T	Vidyant et al 2019 [62]	Indian	No link to HIV infection	No link to stage progression

TLR4	rs4986790 rs4986791	Willie et al 2014 [55]	North America, Caucasian, AA	No association in either racial groups, associate with HIV status	NA
TLR4	rs7873784	Willie et al 2014 [55]	North America, Caucasian, AA	SA in Afroamericans	NA
TLR4	rs10759932	Willie et al 2014 [55]	North America, Caucasian, AA	SA in Caucasians	NA
TLR4	AGCACGG	Willie et al 2014 [55]	North America, Caucasian, AA	SA in HIV infection in Caucasians	NA
TLR4	rs4986791, G allele	Kim et al 2020 [64]	Caucasian and Asian	Strong association to HIV infection	Allele G significant risk factor
TLR4	rs4986790 rs4986791	Tarancon-Diez et al 2018 [67]	Spanish	Asp299Gly independently associated with the occurrence of CVDs in HIV-infected patients.	The proinflammatory profile could be involved in the development of atherosclerosis
TLR4	rs4986790 896A/G	Beima-Sofie et al 2013 [59]	Kenyan, infants	NA with HIV infection	Not associated with disease progression
TLR4	rs4986790 896A/G	Bochud et al 2007 [78]	Swiss	NA with HIV infection	Not associated with disease progression
TLR4	rs4986790 rs4986791 rs7873784	Varshney et al 2022 [82]	Various	association between TB disease in a particular ethnic population	The protection against TB
TLR6	rs5743810 rs5743806 rs1039559 rs3775073	Willie et al 2014 [55]	North America, Caucasian	SA with HIV status, in Casucasians, possible protective effect of rs5743810	NA
TLR6	rs3796508 G/A	Onyishi et al 2021 [60]	various studies	higher risk of cryptococcal meningitis in HIV-negative patients	

TLR6	rs3804099 C/T	Onyishi et al 2021 [60]	various studies	higher risk of cryptococcal meningitis in HIV-negative patients	associated with cerebrospinal fluid cytokine expression
TLR6	rs5743810	Varshney et al 2022 [82]	various	association between TB disease in a particular ethnic population	The protection against TB
TLR7	rs179008	Shi et al 2020 [54]	various	Associated with HIV infection, T allele provides risk effect for infection	NA
TLR7	rs179008, A/A	Said et al 2014 [68]	Omani	Associated with HIV acquisition	Increased VL and high CD4T cell count
TLR7	rs179008, A/A	Anokhin et al 2016 [69]	Tatarstan, Russia	Significant correlations	Increased VL and altered CD4T cell count
TLR7	rs179008, T allele	Oh et al 2009 [72]	German	Associated to HIV-1	T allele linked to high VL, low CD4, faster progression, advanced immune supression
TLR7	rs179008, TC, CC	Valverde-Villegas et al 2017 [80]	Brazilians - European and African ancestry	Not associated to HIV-1 infection	NA
TLR7	rs179008	Singh et al 2020 [73]	West Indian, Naive to ART	NS	TT genotype could protect against the advancement of HIV in early disease
TLR7	rs179008, A/T	Shaikh et al 2019 [71]	Indian	NS	NS
TLR7	rs179008, A/T	Beima-Sofie et al 2013 [59]	Kenyan, infants	NA	Trend for association to mortality in female infants
TLR7	rs179009, A/G	Zhang at al 2020 [70]	Chinese, MSM	NS for acute infection, allele G and CTG haplotype	Higher set point and rapid progression for allele A

				associated with chronic infection	
TLR7	rs179010, C/T rs2074109, T/C rs179009 A/G	Zhang at al 2020 [70]	Chinese, MSM	TTA haplotype associated to lower susceptability in acute HIV infection, but not in chronic	AHI patients with TTA haplotype linked to slower disease progression and lower viral loads
TLR7	rs2074109, G allele	Shaikh et al 2019 [71]	Indian	Associated with HIV infection, G allele less likely to associate with HIV, predisposition factor for HIV	NS
TLR7	rs179009, T/C	Shaikh et al 2019 [71]	Indian	NS	NS
TLR7	rs179009 A/G	Singh et al 2020 [73]	West Indian, Naive to ART	NS, lower risk of HIV-1 infection for AG and AG-GG	A/G genotype could protect the advancement of HIV in advanced disease
TLR7	rs179010 C/T	Zhang at al 2020 [70]	Chinese, MSM	Allele T associated with decreased susceptibility	Allele T associated with slow progression in acute HIV infection
TLR7	rs2074109 T/C	Zhang at al 2020 [70]	Chinese, MSM	NS for acute HIV infection	NA
TLR7	rs1634319	Beima-Sofie et al 2013 [59]	Kenyan, infants	NA	VL in female infants infected by 12 months, VL NS in female infants infected by 1 month, VL NS in male infants
TLR8	rs2159377 354C/T	Willie et al 2014 [55]	North America, Caucasian, AA	Associated with HIV status, SA in AA, potentialy protective against HIV in AA	NA

TLR8	rs3764880	Valverde-Villegas et al 2017 [80]	South Brazil - European and African descendants	Not associated to HIV-1 infection	NA
TLR8	rs3764880, rs2407992	Willie et al 2014 [55]	North America, Caucasian, AA	SA with HIV status, SA in Casucasians	NA
TLR8	rs3764880 A/G	Beima-Sofie et al 2013 [59]	Kenyan, infants	SA with reduced progression	higher VL in female infants, NS of VL in male infants
TLR9	1174 C/T	Jabłońska et al 2020 [66]		Associated with the risk of infectious mononucleosis	Related to the course of acute EBV infection
TLR9	rs187084 1486C/T	Joshi et al 2019 [76]	American, mostly Hispanic	NS	Link to disease progression, weak correlation to lower CD4 count, higher CD8 and IP10 levels
TLR9	rs352140,G/G genotype	Shi et al 2020 [54]	various	May associate with increased risk of HIV infection	NA
TLR9	rs352140 1635 A/G	Kaushik et al 2022 [59]	North Indian	Increased frequenca of A allele in active TB development of naive HIV+ patients	NA
TLR9	rs5743836, C allele	Valverde-Villegas et al 2017 [80]	South Brazil-European and African descendants	C allele more susceptible to HIV in European descendants. C/T genotype protective against HIV-1 in African descendants	NA
TLR9	rs352140	Valverde-Villegas et al 2017 [80]	South Brazil-European and African descendants	NA to HIV-1 infection. A/A genotype higher in the HIV+/HCV+ group in African descendants	NA

TLR9	rs352140 1635 G/G, A/G	Said et al 2014 [68]	Indian	Associated with HIV acquisition	GG associated with lower CD4 T-cell count. AG related to higher CD4 T- cell count
TLR9	rs352140 1635 A/G	Shaikh et al 2019 [71]	Indian	NS	Less likely to associate with HIV progression, allele A associated with slow disease progression
TLR9	rs352140 1635A/G, 1174G/A	Bochud et al 2007 [78]	Swiss	NA	Associated with rapid progression, not with slow progressors
TLR9	rs352140 1635A/G	Joshi et al 2019 [76]	Americans, mostly Hispanic	NA	Link to disease progression (higher VL and faster progression)
TLR9	rs352140 1635A/A	Vallejo et al 2020 [77]	Spanish	NA	Associated with higher HIV rebound after ART interruption, may impact outcomes
TLR9	rs352140 2848 G/A	Kulmann-Leal et al 2022 [79]	Brazilians	Potential influence on viral infection	GG genotype serving as protective factor for non-Caucasians
TLR9	rs352140 2848 C/T	Jabłońska et al 2021 [81]	Polish, children and adolescents	Assocation with CMV infected HIV/CMV co-infected subjects	CMV DNAemia among HIV-infected patients with CMV
TLR9	rs5743836 -1237T/C	Varshney et al 2022 [82]	various	The protection against TB	The protection against TB linked

Abbreviations: ART- antiretroviral treatment, CMV- cytomegalovirus, CVDs- cardiovascular diseases, EBV- Epstein-Barr virus, HIV- human immunodeficiency virus, MSM- men who have sex with men, NA- not available, NS- non significant, SA- significant association, SNP- single nucleotide polymorphism, TB- tuberculosis, VL- viral load
